# Supplementary material for: Knockdown of a regulatory barcode shifts macrophage polarization destination from M1 to M2 and increases pathogen burden upon S. aureus infection
Source: eLife. 2026 Nov 2;12:RP91547. doi: 10.7554/eLife.91547 (PMC13727075; doi:10.7554/eLife.91547)
Supplement: Supplementary file 1 [file elife-91547-mdarchecklist1.docx]

**Materials Design Analysis Reporting (MDAR) Checklist**

*eLife — Author Submission Template*

The MDAR framework establishes a minimum set of requirements for transparent reporting in the life sciences. eLife asks authors to provide detailed information within their article to facilitate the interpretation and replication of their work. *For each item that applies, indicate where in the article the information is provided (section name or figure legend). If an item is not applicable to your study, tick the N/A column.*

Authors may also upload supporting materials to comply with relevant reporting guidelines for health-related research (EQUATOR Network), life science research (BioSharing), or animal research (ARRIVE Guidelines / STRANGE Framework).

| **Manuscript Title:** |  |
| --- | --- |
| **Corresponding Author:** |  |
| **Date:** |  |

| **Requirement** | **Indicate where provided in article (section / figure legend)** | **N/A (tick if not applicable)** |
| --- | --- | --- |
| **MATERIALS** | | |
| ***Newly Created Materials*** | | |
| The manuscript includes a dedicated materials availability statement disclosing availability of newly created materials, how they can be accessed, and any restrictions. |  | NA |
| ***Antibodies*** | | |
| For commercial reagents: provide supplier name, catalogue number, and RRID (if available). |  | NA |
| ***DNA and RNA Sequences*** | | |
| Short novel DNA/RNA including primers and probes: sequences included in manuscript or deposited in a public repository. | *Included in Key Resource Table and Supplementary File* |  |
| ***Cell Materials*** | | |
| Cell lines: provide species, strain, accession number OR supplier name, catalogue number, clone number, OR RRID. | *Included in Key Resource Table and Material and Methods Section* |  |
| Primary cultures: provide species, strain, sex of origin, genetic modification status. |  | NA |
| ***Experimental Animals*** | | |
| Laboratory animals / model organisms: provide species, strain, sex, age, genetic modification status, and supplier/RRID. |  | NA |
| Animals observed in or captured from the field: provide species, sex, and age where possible. |  | NA |
| ***Plants and Microbes*** | | |
| Plants: provide species, strain, ecotype, cultivar where relevant, unique accession number, and source. |  | NA |
| Microbes: provide species, strain, unique accession number, and source. |  | NA |
| ***Human Research Participants*** | | |
| Report age, sex, gender, and ethnicity for all study participants (within privacy constraints). State if these demographics were not collected. |  | NA |
| **DESIGN** | | |
| ***Study Protocol*** | | |
| If the study protocol was pre-registered, provide DOI. For clinical trials, provide trial registration number or cite DOI. | *e* | NA |
| ***Laboratory Protocol*** | | |
| Provide DOI or other citation details if detailed step-by-step protocols are publicly available. |  | NA |
| ***Experimental Study Design (In Vivo Studies)*** | | |
| Sample size determination: state whether and how an appropriate sample size was determined. |  | NA |
| Randomisation: state whether and how samples/animals were randomised to groups. |  | NA |
| Blinding: state whether investigators were blinded during data collection and/or analysis. |  | NA |
| Inclusion / exclusion criteria: state pre-established criteria for including or excluding subjects. |  | NA |
| ***Sample Definition and In-Laboratory Replication*** | | |
| State the number of times each experiment was independently replicated. Define whether data describe technical or biological replicates. | *Figure legends* |  |
| ***Ethics*** | | |
| Studies involving human participants: state details of authority granting ethics approval (IRB or equivalent) and reference number. |  | NA |
| Studies involving experimental animals: state details of authority granting ethics approval (IACUC or equivalent) and reference number. |  | NA |
| Studies involving specimen / field samples: state if relevant permits were obtained; provide details of approving authority. If none required, explain why. |  | NA |
| ***Dual Use Research of Concern (DURC)*** | | |
| If the study is subject to DURC regulations, state the authority granting approval and reference number. |  | NA |
| **ANALYSIS** | | |
| ***Attrition*** | | |
| Describe whether exclusion criteria were pre-established. Report if any samples or data points were omitted from analysis, and provide justification. |  | NA |
| ***Statistics*** | | |
| Describe statistical tests used and justify the choice of tests. Report exact values of N, definitions of centre, dispersion/precision measures (mean, SD, SEM, CI), and effect sizes for major results. Report exact p-values wherever possible. | *Material and Methods – Statistical Analysis / Figure legends* |  |
| ***Data Availability*** | | |
| The manuscript includes a data availability statement providing details for access to newly created and reused datasets (or notes restrictions on access). |  |  |
| When newly created datasets are publicly available: provide accession number, DOI, and licensing details. |  | NA |
| If reused data are publicly available: provide accession number, DOI, URL, or citation. | *e.g. PDB ID; public repository URL* |  |
| ***Code Availability*** | | |
| For any computer code, software, or mathematical algorithms essential for replicating main findings: the manuscript includes a statement providing details for access or notes restrictions. | *“Code availability” section* |  |
| Where newly generated code is publicly available: provide accession number, DOI, URL, and licensing details. |  |  |
| **REPORTING** | | |
| ***Adherence to Community Standards*** | | |
| State whether relevant discipline-specific reporting guidelines have been followed (e.g., ICMJE, MIBBI, ARRIVE, STRANGE, CONSORT, PRISMA) and whether the corresponding checklist is provided with the manuscript. | *e.g. Methods; checklist uploaded as supplementary file* |  |

**Statistics guidance (eLife):** For each experiment report the statistical tests used, exact N, definitions of centre, dispersion/precision measures (mean, SD, SEM, CI), effect sizes (e.g. Cohen's d, Pearson's r), and exact p-values. Raw data should be shown in figures when N < 10 per group. Describe how samples were allocated to groups and whether masking was applied during collection/analysis.

**Note:** *Data availability and ethics information is also collected separately in the eLife submission form.*
