# Supplementary material for: Knockdown of a regulatory barcode shifts macrophage polarization destination from M1 to M2 and increases pathogen burden upon S. aureus infection
Source: eLife. 2026 Nov 2;12:RP91547. doi: 10.7554/eLife.91547 (PMC13727075; doi:10.7554/eLife.91547)
Supplement: Supplementary file 2 [file elife-91547-supp1.docx]

*I****n vitro stimulant data***. Details on the triggers and the end time point considered for the transcriptome analysis. (hrs – hours)

| **Stimulant(s)** | **Time Point** | **# Samples** |
| --- | --- | --- |
| Baseline (Mϕ) () | 6hrs, 24hrs, 72 hrs | 3;3;7 |
| High Density Lipoprotein (HDL) | 6 hrs | 4 |
| Lauric Acid (LA) | 24 hrs | 6 |
| Linoleic Acid (LiA) | 24 hrs | 6 |
| Oleic Acid (OA) | 24 hrs | 9 |
| Palmitic Acid (PA) | 24 hrs | 9 |
| Stearic Acid (SA) | 24 hrs | 3 |
| Glucocorticoids (GC) | 72 hrs | 3 |
| Standard lipopolysaccharide (sLPS) | 72 hrs | 8 |
| Ultrapure Lipopolysaccharides (upLPS) | 72 hrs | 6 |
| Interferon beta (IFNβ) | 72 hrs | 3 |
| Interferon gamma (IFNγ) | 72 hrs | 7 |
| Interleukin-10 (IL10) | 72 hrs | 3 |
| Interleukin-13 (IL13) | 72 hrs | 3 |
| Interleukin-4 (IL4) | 72 hrs | 10 |
| Pam3CysSerLys4 (P3C) | 72 hrs | 3 |
| Prostaglandin E2 (PGE2) | 72 hrs | 3 |
| P3C + PGE2 | 72 hrs | 3 |
| Tumour Necrosis Factor (TNF) | 72 hrs | 6 |
| sLPS+IC | 72 hrs | 3 |
| sLPS + IFNγ | 72 hrs | 3 |
| IFNγ +TNF | 72 hrs | 3 |
| TPP (TNF + PGE2 +P3C) | 72 hrs | 10 |
| TPP + IFNβ | 72 hrs | 3 |
| TPP + IFNβ + IFNγ | 72 hrs | 3 |
| TNF alpha + P3C | 72 hrs | 3 |

| TNF alpha +PGE2 | 72 hrs | 3 |
| --- | --- | --- |
| IL4 + upLPS | 72 hrs | 3 |
| upLPS +IC | 72 hrs | 3 |
