# Supplementary material for: Knockdown of a regulatory barcode shifts macrophage polarization destination from M1 to M2 and increases pathogen burden upon S. aureus infection
Source: eLife. 2026 Nov 2;12:RP91547. doi: 10.7554/eLife.91547 (PMC13727075; doi:10.7554/eLife.91547)
Supplement: Supplementary file 3 [file elife-91547-supp2.docx]

# **The list of primers used for the qRT-PCT**

| **Gene** | **Primer** | **Sequence** |
| --- | --- | --- |
| CEBPB | Forward | ATGTTCCTACGGGCTTGTTG |
|  | Reverse | CCCAAAAGGCTTTGTAACCA |
| NFE2L2 | Forward | GCGACGGAAAGAGTATGAGC |
|  | Reverse | GTTGGCAGATCCACTGGTTT |
| BCL3 | Forward | CCCTATACCCCATGATGTGC |
|  | Reverse | GGTGTCTGCCGTAGGTTGTT |
| STAT4 | Forward | TACCTAATGCTTGGGCATCC |
|  | Reverse | TCTGCCAGCATATGGAGTTG |
| IL1B | Forward | ATGATGGCTTATTACAGTGGCAA |
|  | Reverse | GTCGGAGATTCGTAGCTGGA |
| IL10 | Forward | TGCCTTCAGCAGAGTGAAGA |
|  | Reverse | TGGGTCTTGGTTCTCAGC |
| ARG1 | Forward | ACAGTTTGGCAATTGGAAGCA |
|  | Reverse | CACCCAGATGACTCCAAGATCAG |
| MRC1 | Forward | GGGTTGCTATCACTCTCTATGC |
|  | Reverse | TTTCTTGTCTGTTGCCGTAGTT |
| TGFB | Forward | CTAATGGTGGAAACCCACAACG |
|  | Reverse | TATCGCCAGGAATTGTTGCTG |
| CXCL2 | Forward | CGCCCATGGTTAAGAAAATCA |
|  | Reverse | CCTTCTGGTCAGTTGGATTTGC |
| iNOS | Forward | CAGCGGGATGACTTTCCAA |
|  | Reverse | AGGCAAGATTTGGACCTGCA |
| SOCS3 | Forward | GTCACCCACAGCAAGTTT |
|  | Reverse | CTGAGCGTGAAGAAGTGG |
