# Supplementary material for: Knockdown of a regulatory barcode shifts macrophage polarization destination from M1 to M2 and increases pathogen burden upon S. aureus infection
Source: eLife. 2026 Nov 2;12:RP91547. doi: 10.7554/eLife.91547 (PMC13727075; doi:10.7554/eLife.91547)
Supplement: Supplementary file 4 [file elife-91547-supp3.docx]

**The list of clusters, associated members, network details of regulatory cores and the number of TFs in the barcodes.** * corresponds to the clusters containing a single member. Nodes correspond to the number of genes and edges correspond to the number of interactions in the network.

| **Cluster ID** | **Cluster members** | **Core Network statistics (Nodes**  **;** | **No. of Tfs in the Cluster centric**  **transcription factor based barcode** |
| --- | --- | --- | --- |

|  |  | **Edges)** |  |
| --- | --- | --- | --- |
| Cluster 1 | P3C, P3C+PGE2,  PGE2 | 2427; 2747 | 79 |
| Cluster 2* | IL4+upLPS | 4959; 6498 | 78 |
| Cluster 3 | IL13, IL4 | 1829; 1970 | 42 |
| Cluster 4 | GC, IL10 | 289; 302 | 5 |

| Cluster 5 | upLPS, upLPS+IC | 1576; 1698 | 43 |
| --- | --- | --- | --- |
| Cluster 6 | HDL, IFNβ | 901; 992 | 66 |
| Cluster 7 | TNFα, IFNγ+TNFα,  sLPS+IFNγ | 364; 376 | 31 |
| Cluster 8 | TPP+IFNβ,  TPP+IFNβ+IFNγ | 3103; 3432 | 113 |
| Cluster 9 | TNFα+P3C,  TNFα+PGE2 | 3812; 4172 | 87 |
| Cluster 10 | OA, LiA, LA | 1922; 2059 | 30 |
| Cluster 11 | sLPS, sLPS+IC, TPP | 2028; 2145 | 52 |
| Cluster 12* | IFNγ | 5268; 6387 | 42 |
| Cluster 13 | PA, SA | 3482; 3945 | 68 |
