# Supplementary material for: Knockdown of a regulatory barcode shifts macrophage polarization destination from M1 to M2 and increases pathogen burden upon S. aureus infection
Source: eLife. 2026 Nov 2;12:RP91547. doi: 10.7554/eLife.91547 (PMC13727075; doi:10.7554/eLife.91547)
Supplement: Peer review history

# Editor report: eLife Assessment

**DOI:** 10.7554/eLife.91547.3.sa0

**Authors:** Jalees Rehman

**Affiliations:** University of Illinois Chicago United States

**Keywords:** Solid, Valuable

The authors of this manuscript study the transcriptional regulators that allow macrophages to assume different functional phenotypes in response to immune stimuli. They generate a computational map of the gene regulatory networks involved in determining macrophage phenotypes and experimentally validate the role of putative regulatory factors in a myeloid cell line. This study represents a **valuable** approach to understanding how gene regulation impacts macrophage polarization and their conclusions are supported by **solid** computational and experimental evidence. The revision has clarified that the focus is the identification of the regulatory barcodes in a myeloid cell line. Future studies in primary cells and in vivo will be required to assess the roles of these regulators in a broader context.

# Referee report: Reviewer #1 (Public Review)

**DOI:** 10.7554/eLife.91547.3.sa1

Summary:

Ravichandran et al investigate the regulatory panels that determine the polarization state of macrophages. They identify regulatory factors involved in M1 and M2 polarization states by using their network analysis pipeline. They demonstrate that a set of three regulatory factors (RFs) i.e., CEBPB, NFE2L2, and BCL3 can change macrophage polarization from the M1 state to the M2 state. They also show that siRNA-mediated knockdown of those 3-RF in THP1-derived M0 cells, in the presence of M1 stimulant increases the expression of M2 markers and showed decreased bactericidal effect. This study provides an elegant computational framework to explore the macrophage heterogeneity upon different external stimuli and adds an interesting approach to understanding the dynamics of macrophage phenotypes after pathogen challenge.

Strengths:

This study identified new regulatory factors involved in M1 to M2 macrophage polarization. The authors used their own network analysis pipeline to analyze the available datasets. The authors showed 13 different clusters of macrophages that encounter different external stimuli, which is interesting and could be translationally relevant as in physiological conditions after pathogen challenge, the body shows dynamic changes in different cytokines/chemokines that could lead to different polarization states of macrophages. The authors validated their primary computational findings with in vitro assays by knocking down the three regulatory factors-NCB.

# Referee report: Reviewer #2 (Public Review)

**DOI:** 10.7554/eLife.91547.3.sa2

Summary:

The authors of this manuscript address an important question regarding how macrophages respond to external stimuli to create different functional phenotypes, also known as macrophage polarization. Although this has been studied extensively, the authors argue that the transcription factors that mediate the change in state in response to a specific trigger remain unknown. They create a "master" human gene regulatory network and then analyze existing gene expression data consisting of PBMC-derived macrophage response to 28 stimuli, which they sort into thirteen different states defined by perturbed gene expression networks. They then identify the top transcription factors involved in each response that have the strongest predicted association with the perturbation patterns they identify. Finally, using *S. aureus* infection as one example of a stimulus that macrophages respond to, they infect THP-1 cells while perturbing regulatory factors that they have identified and show that these factors have a functional effect on the macrophage response.

Strengths:

The computational work done to create a "master" hGRN, response networks for each of the 28 stimuli studied, and the clustering of stimuli into 13 macrophage states is useful. The data generated will be a helpful resource for researchers who want to determine the regulatory factors involved in response to a particular stimulus and could serve as a hypothesis generator for future studies.

The streamlined system used here - macrophages in culture responding to a single stimulus - is useful for removing confounding factors and studying the elements involved in response to each stimulus.

The use of a functional study with *S. aureus* infection is helpful to provide proof of principle that the authors' computational analysis generates data that is testable and valid for in vitro analysis.

[Reviewing Editor comments on revised version: the authors have made minimal changes and we have made a modest modification to the eLife Assessment, without returning the revised version to the original reviewers.]

# Author comment: Author response

**DOI:** 10.7554/eLife.91547.3.sa3

**Authors:** Sathyabaarathi Ravichandran, Bharath Bhatt, Awantika Shah, Debajyoti Das, Kithiganahalli K Narayanaswamy Balaji, Nagasuma Chandra

**Affiliations:** Indian Institute of Science Bangalore India; Indian Institute of Science Bangalore Bangalore India; Indian Institute of Science Bangalore Bangalore India; Indian Institute of Science Bangalore Bangalore India; Indian Institute of Science Bangalore Bangalore India; Department of Biochemistry, Indian Institute of Science Bangalore India

The following is the authors’ response to the original reviews.

**Reviewer #1 (Public Review):**

Summary:

Ravichandran et al investigate the regulatory panels that determine the polarization state of macrophages. They identify regulatory factors involved in M1 and M2 polarization states by using their network analysis pipeline. They demonstrate that a set of three regulatory factors (RFs) i.e., CEBPB, NFE2L2, and BCL3 can change macrophage polarization from the M1 state to the M2 state. They also show that siRNA-mediated knockdown of those 3-RF in THP1-derived M0 cells, in the presence of M1 stimulant increases the expression of M2 markers and showed decreased bactericidal effect. This study provides an elegant computational framework to explore the macrophage heterogeneity upon different external stimuli and adds an interesting approach to understanding the dynamics of macrophage phenotypes after pathogen challenge.

Strengths:

This study identified new regulatory factors involved in M1 to M2 macrophage polarization. The authors used their own network analysis pipeline to analyze the available datasets. The authors showed 13 different clusters of macrophages that encounter different external stimuli, which is interesting and could be translationally relevant as in physiological conditions after pathogen challenge, the body shows dynamic changes in different cytokines/chemokines that could lead to different polarization states of macrophages. The authors validated their primary computational findings with in vitro assays by knocking down the three regulatory factors-NCB.

We thank the reviewer for reading our manuscript and for the encouraging comments.

Weaknesses:

One weakness of the paper is the insufficient analysis performed on all the clusters. They used macrophages treated with 28 distinct stimuli, which included a very interesting combination of pro- and anti-inflammatory cytokines/factors that can be very important in the context of in vivo pathogen challenge, but they did not characterize the full spectrum of clusters.

We have performed a functional enrichment analysis of all the clusters and added a section describing the results (Fig 1B). We believe this work will provide a basis for future experiments to characterize other clusters.

We have also performed a Principal Component Analysis (PCA) using hall mark genes of inflammation and the NCB panel alone to show the relative position of all clusters with respect to each other

Although they mentioned that their identified regulatory panels could determine the precise polarization state, they restricted their analysis to only the two well-established macrophage polarization states, M1 and M2. Analyzing the other states beyond M1 and M2 could substantially advance the field. They mentioned the regulatory factors involved in individual clusters but did not study the potential pathway involving the target genes of these regulatory factors, which can show the importance of different macrophage polarization states. Importantly, these findings were not validated in primary cells or using in vivo models.

We agree it would be useful to demonstrate the polarization switch in other systems as well. However, it is currently infeasible for us to perform these experiments.

**Reviewer #2 (Public Review):**

Summary:

The authors of this manuscript address an important question regarding how macrophages respond to external stimuli to create different functional phenotypes, also known as macrophage polarization. Although this has been studied extensively, the authors argue that the transcription factors that mediate the change in state in response to a specific trigger remain unknown. They create a "master" human gene regulatory network and then analyze existing gene expression data consisting of PBMC-derived macrophage response to 28 stimuli, which they sort into thirteen different states defined by perturbed gene expression networks. They then identify the top transcription factors involved in each response that have the strongest predicted association with the perturbation patterns they identify. Finally, using *S. aureus* infection as one example of a stimulus that macrophages respond to, they infect THP-1 cells while perturbing regulatory factors that they have identified and show that these factors have a functional effect on the macrophage response.

Strengths:

The computational work done to create a "master" hGRN, response networks for each of the 28 stimuli studied, and the clustering of stimuli into 13 macrophage states is useful. The data generated will be a helpful resource for researchers who want to determine the regulatory factors involved in response to a particular stimulus and could serve as a hypothesis generator for future studies.

The streamlined system used here - macrophages in culture responding to a single stimulus - is useful for removing confounding factors and studying the elements involved in response to each stimulus.

The use of a functional study with *S. aureus* infection is helpful to provide proof of principle that the authors' computational analysis generates data that is testable and valid for in vitro analysis.

We thank the reviewer for reading our manuscript and for the encouraging comments

Weaknesses:

Although a streamlined system is helpful for interrogating responses to a stimulus without the confounding effects of other factors, the reality is that macrophages respond to these stimuli within a niche and while interacting with other cell types. The functional analysis shown is just the first step in testing a hypothesis generated from this data and should be followed with analysis in primary human cells or in an in vivo model system if possible.

It would be helpful for the authors to determine whether the effects they see in the THP-1 immortalized cell line are reproduced in another macrophage cell line, or ideally in PBMC-derived macrophages.

We agree; It would be useful in the future to demonstrate the polarization switch in other systems as well. We believe the results we provide here will inform future studies on other systems.

The paper would benefit from an expanded explanation of the network mining approach used, as well as the cluster stability analysis and the Epitracer analysis. Although these approaches may be published elsewhere, readers with a non-computational background would benefit from additional descriptions.

We have elaborated on the network mining approach and added a schematic diagram (Fig S13) to describe the EpiTracer algorithm.

Although the authors identify 13 different polarization states, they return to the iM0/M1/M2 paradigm for their validation and functional assays. It would be useful to comment on the broader applications of a 13-state model.

We have included a new figure panel describing the functional enrichment analysis of all the clusters (Fig 1B) and added a section describing the results. We have also performed a Principal Component Analysis (PCA) using hallmark gene of inflammation and the NCB panel alone to show the relative position of all clusters with respect to each other. The PCA plot shows that C11(M1) and C3(M2) are roughly at two extreme ends, with other clusters between them, forming something resembling a punctuated continuum of states.

The relative contributions of each "switching factor" to the phenotype remain unclear, especially as knocking out each individual factor changes different aspects of the model (Fig. S5).

Fig S5 shows the effect on phenotype upon individual knockdown of the switching factors, from which we deduce that CEBPB has the largest contribution in determining the phenotype. However, we maintain that all three genes are necessary as a panel for M1/M2 switching.

**Reviewer #1 (Recommendations For The Authors):**

The manuscript by Ravichandran et al describes the networks of genes that they named j"RF" associated with M1 to M2 polarization of macrophages by using their computational pipelines. They have shown 13 clusters of human macrophage polarization state by using an available database of different combinatorial treatments with cytokines, endotoxin, or growth factors, which is interesting and could be useful in the research field. However, there are a few comments which will help to understand the subject more precisely.

(1,2) The authors claimed to identify key regulatory factors involved in the human macrophage polarization from M1 to M2. However, recent advances suggest that macrophage polarization cannot be restricted to M1 and M2 only, which is also supported by the authors' data that shows 13 clusters of macrophages. However, they only focused on the difference between clusters 11 and 3 considering conventional M1 and M2. It will be more interesting to analyze the other clusters and how they relate to the established and simplistic M1 and M2 paradigms.

It will be interesting to know if they found any difference in the enriched pathways among these different clusters considering the exclusive regulatory factors and their targets.

We appreciate the point and have addressed it as follows. In the revised manuscript, we have discussed the clusters in detail and have provided the key regulatory factors (RF) combinations and target genes that define distinct macrophage population states (Please refer: Data file S2, S3). We have also discussed the associated immunological processes with each cluster, particularly in relation to the C11 and C3 clusters. We have added a new panel in Fig 1 to illustrate a heatmap indicating the enrichment of pathways relevant to inflammation in each of the clusters (Fig 1B). Indeed, there is a substantial difference in the enrichment terms between the extreme ends (M1, M2) and significant differences in some of the pathways between clusters.

(3) The authors have shown the involvement of NCB at 72h post LPS treatment. Are these RF involved in late response genes or act at the earlier time point of LPS treatment? Understanding the RF involvement in the dynamic response of macrophages to any stimulant will be important.

Using the data available for different time points (30 mins to 72 hours), we plotted the fold change (with respect to unstimulated cells) in M1 and M2 clusters for each of the NCB genes and observe clear divergence in the trend at 24 hours and have provided them as newly added (Supplementary Figure 9 A, B, C).

(4) The authors showed that the knockdown of RF- NCB can switch the M1 to M2. However, they showed a few conventional markers known to be M2 markers. What happens if NCB is overexpressed or knocked down in other treatment conditions/other clusters? Is the RF-NCB only involved in these two specific stimulations or their overexpression can promote M2 polarization in any given stimuli?

It is an interesting question but for practical reasons, experimental work was limited to M1 and M2 clusters as the aim was to establish proof of concept and could not be scaled up for all clusters, which would require a large amount of work and possibly a separate study. We believe the description of the clusters that we have provided will enable the design of future experiments that will throw light on the significance of the intermediate clusters.

(5) The authors have shown that knockdown of RF- NCB decreases pathogen clearance, but what are their altered functions? Are they more efficient in cellular debris clearance or resolution of inflammation? The authors can check the mRNA expression of markers/cytokines involved in those processes, in the NCB knockdown condition.

Indeed. Expression levels were measured for the following genes: CXCL2, IL1B, iNOS, SOCS3 (which are pro-inflammatory markers), as well as MRC1, ARG1, TGFB, IL10 (anti-inflammatory markers), as shown in Fig 4B.

Minor comments:

(1, 2). How the authors evaluate the performance of their knowledge-based gene network. The authors should write the methods in detail, how they generated the simulated network, and evaluated the simulated dataset.

Gene network construction and module detection have many tools available. The authors need to mention which one they used. The authors should show whether their findings are consistent with at least another two module-detection methods (eg; "RedeR") to strengthen their claim.

We have added a schematic figure (Supplementary Fig S11) and detailed description of network construction and mining in the Methods section, as follows: We have reconstructed a comprehensive knowledge-based human Gene Regulatory Network (hGRN), which consists of Regulatory Factors (RF) to Target Gene (TG) and RF to RF interactions. To achieve this, we curated experimentally determined regulatory interactions (RF-TG, RF-RF) associated with human regulatory factors (Wingender et al., 2013). These interactions were sourced from several resources, including: (a) literature-curated resources like the Human Transcriptional Regulation Interactions database (HTRIdb) (Bovolenta et al., 2012), Regulatory Network Repository (RegNetwork) (Liu et al., 2015), Transcriptional Regulatory Relationships Unraveled by Sentence-based Text-mining (TRRUST) (Han et al., 2015), and the TRANSFAC resource from Harmonizome (Rouillard et al., 2016); (b) ChEA3, which contains ChIP-seq determined interactions (Keenan et al., 2019); and (c) high-confidence protein-protein binding interactions (RF-RF) from the human protein-protein interaction network-2 (hPPiN2) (Ravichandran et al., 2021). As a result, our hGRN comprises 27,702 nodes and 890,991 interactions. It is important to note that none of the edges/interactions in the hGRN are data-driven. We utilized this extensive hGRN, which encompasses the experimentally determined interactions/edges, to infer stimulant-specific hGRNs and top paths using our in-house network mining algorithm, ResponseNet. We have previously demonstrated that ResponseNet, which utilizes a knowledge-based network and a sensitive interrogation algorithm, outperformed data-driven network inference methods in capturing biologically relevant processes and genes, whose validation is reported earlier (Ravichandran and Chandra, 2019; Sambaturu et al., 2021).

We utilized our in-house response network approach to identify the stimulant-specific top active and repressed perturbations (Ravichandran and Chandra, 2019; Sambaturu et al., 2021). This is clearly described in the revised manuscript. To summarize, we generated stimulant-specific Gene Regulatory Networks (GRNs) by applying weights to the master human Gene Regulatory Network (hGRN) based on differential transcriptomic responses to stimulants (i.e., comparing stimulant-treated conditions to baseline). We then produced individually weighted networks for each stimulant and implemented a refined network mining technique to extract the most significant pathways. Furthermore, we have previously conducted a systematic comparison of our network mining strategy with other data-driven module detection methods, including jActiveModules (Ideker et al, 2002), WGCNA (Langfelder et al, 2008), and ARACNE (Margolin et al, 2006). Our findings demonstrated that our approach outperformed conventional data-driven network inference methods in capturing the biologically pertinent processes and genes (Ravichandran and Chandra, 2019). Since we have experimentally validated what we predicted from the network analysis, we do not see a need for performing the computational analysis with another algorithm. Moreover, different network analyses are based on different aspects of identifying functionally relevant genes or subnetworks. While each of them output useful information, given the scale of the network and the number of different biologically significant subnetworks and genes that could be present in an unbiased network such as what we have used, the output from different methods need not agree with each other as they may capture different aspects all together and hence is not guaranteed to be informative.

(3) Representation of Fig 2B is difficult to understand the authors' interpretation of 'the 3-RF combination has 1293 targets, 359 covering about 53% of the top-perturbed network' for general readers. If the authors can simplify the interpretation will be helpful for the readers.

This is replaced with clearer figures in the revised manuscript (Figure 2A, 2B), and the associated text is also rephrased for clarity.

**Reviewer #2 (Recommendations For The Authors):**

Major comments:

(1) It would be helpful for the authors to determine whether the effects they see in the THP-1 immortalized cell line are reproduced in another macrophage cell line, or ideally in PBMC-derived macrophages if this is feasible. If using PBMC- or bone marrow-derived macrophages is beyond the scope of what the authors can reasonably perform, they could consider using another macrophage cell line such as RAW 264.7 cells, which would also provide orthogonal validation from a mouse model.

At this point of time, it is unfortunately infeasible for us to perform these experiments, due to resource limitation. Moreover, it would require a lot of time. We hope that our work provides pointers for anyone working on mouse models or other model systems to design their studies on regulatory controls and the aspect of generalizability of our findings in Thp-1 cell lines to other systems will eventually emerge.

(2) It would be helpful for the authors to provide an expanded explanation of the network mining approach used, as well as the cluster stability analysis and the Epitracer analysis. Although these approaches may be published elsewhere, readers with a non-computational background would benefit from additional descriptions. A schematic figure would also be helpful to clarify their approach.

We have added a new schematic diagram in Supplementary figures (S13) and a detailed text in the Methods section describing the network mining analysis and epitracer identification in the revised manuscript.

(3) It would be helpful for the authors to comment on whether the thirteen polarization states that they identify align with other analyses that have been performed using data collected from stimulated macrophages, or whether this is a novel finding, especially as the original paper from which the primary data are derived identified 9 clusters. More broadly, since the authors eventually return to the M1-M2 paradigm, it is unclear whether there is any functional support for a 13-state model - it is also possible that macrophages exist along a continuum of stimulation states rather than in discrete clusters. This at least merits further discussion, which could focus on different axes of polarization as discussed and shown in the original paper.

As described in the manuscript, Clustering based on the differential transcriptome profile of RF-set1, which contains 265 transcription factors (TFs), in response to 28 stimulants, resulted in 13 distinct clusters. The cluster member associations inferred from RF-set1 were similar in number and pattern to those inferred from the entire differential transcriptome (n=12,164; Fig. S2, cophenetic coefficient = 0.68; p-value = 1.25e−51). Furthermore, the inferred cluster pattern largely matched the clustering pattern previously described for the same dataset (Xue et al., 2014). Our contribution: The pattern we observed from the top-ranked epicenters in each cluster suggests that a subset of differentially expressed genes (DEGs) present in our top networks is sufficient for achieving differentiation. Our gene-regulatory models suggest that saturated (SA and PA) and unsaturated (LA, LiA, and OA) fatty acids, which were previously grouped together, mediate distinct modes of resolution and are now separated into two sub-branches. Similarly, the effects of IFNγ and sLPS, previously combined, are now distinctly resolved, aligning with known regulatory differences (Hoeksema et al., 2015; Kang et al., 2019).

The principal takeaway from this analysis is not the exact number of clusters but rather the molecular basis it provides for the differentiation of functional states, with M1 and M2 representing two ends of the spectrum. Several other states are dispersed within the polarization spectrum, which we describe as a punctuated continuum. For our switching studies, we focused on clusters C11 (M1-like) and C2 (M2-like) due to their established functional relevance. However, future studies are required to explore the functional relevance of other clusters. We have added a discussion on this aspect as suggested.

(4) It would be helpful to define the contribution of each component of the NCB group to M1 polarization.

We assessed the impact of CEBPB, NFE2L2, and BCL3 on C2 (M1-like) polarization states by quantifying the expression levels of M1 and M2 markers. Our findings indicate that knocking down CEBPB led to a significant downregulation in the expression of M1 markers and an increase in M2 marker expression. In contrast, NFE2L2 and BCL3 knockdown resulted in decreased expression of M1 markers without a corresponding significant increase in M2 markers. These results suggest that CEBPB is crucial for M1 to the M2 transition. We have added a note on pg 22 to emphasize this better.

(5) NRF2, CEBPb, and BCL3 all have well-described roles in macrophage polarization. To add clarity to their discussion, the authors should cite relevant literature (eg PMIDs 15465827, 27211851, and others) and discuss how their findings extend what is currently known about the contribution of these individual proteins to macrophage responses.

The role of NFE2L2, CEBPB and BCL3 in macrophage polarization and state transition are described in the discussion section. The PMIDs mentioned by the reviewer are added as well.

(6) The effect size of NCB knockdown in the in vitro Staph aureus model shown in 4C is fairly small - bacterial killing assays typically require at least a log of difference to demonstrate a convincing effect. It would be helpful for the authors to include a positive control for this experiment (for example, STAT4) to frame the magnitude of their effect.

We thank the reviewer for the comment, however, we would like to point out that the difference in CFU plotted in log_10_ scale, as per common practice. The CFUs are therefore almost halved due to the knockdown in absolute scale and reproduced multiple times with statistically significant results (p-value <0.01). We feel it is sufficient to demonstrate that the NCB geneset by themselves bring out a change in polarization and hence the killing effect. We have used STAT4 as a control for marker measurements as shown in Fig 3C. While carrying out CFU with siSTAT4 may add additional information, we have proceeded to perform the infection experiments with and without the NCB knockdown as that remains the main focus of the study.

Minor recommendations:

(1) Is there a difference between the data represented in Figure 1A-B and Figure S1? If this is the same data, there is no need to repeat it, and Figure 1 could be composed only of the current panels C and D.

We have removed Figure1 A and B as it illustrates the same point as Figure S1. We have retained Figures C and D and renamed them as new Figure 1A and C. In addition, we have added a new panel Fig 1B (in response to earlier points).

(2) Could Figure 2B be represented in a different way? The circles do not contain any readable information about the genes, and it may be less visually overwhelming to represent this with just the large and small triangles. Perhaps the individual genes represented by the circles could be listed in a supplemental table or Excel file.

We have provided a new Figure 2 A and B panels for the M1 and M2 clusters respectively, which has only the barcode genes along with a functional annotation. The full network is already provided in supplementary data.

(3) When indicating the N for all experiments performed in the figure legends, the authors should indicate whether these were technical or biological replicates.

We appreciate the reviewers for the suggestion. We have indicated what N is for all figure legends.

(4) Fig 3B: the y-axis is confusing - it appears that normalization is actually to the untreated cells.

Yes indeed. The normalization is with respect to the untreated cells as per standard practice. We have indicated this clearly in the legend.

(5) The 72-hour time point in Fig S8 shows unexpected results. Could the authors explain or propose a hypothesis for why CXCL2 and IL1b abruptly decrease while iNOS and MRC1 abruptly increase?

The purpose of the mentioned experiment was to standardize the time point of M1 polarization post *S. aureus* infection. In this regard, we profiled the expression levels of markers at various time points. We chose to study the 24 hour time point for all the future experiments based on the significant upregulation of NCB seen in the macrophages. We believe that the 72 hour time point may show effects that are different since the initial immune response would have waned leading to differences in cytokine dynamics. However, as this is not the focus of our study, we are not discussing this aspect further.
